# Clinical Observation of the Correlation Between Overactive Bladder and Atopic Constitution in Children

**DOI:** 10.3389/fped.2021.646118

**Published:** 2021-05-07

**Authors:** Yue Zheng, Zhou Zhang, Ling Hou, Xiuli Wang, Kailei Jiang, Shucheng Zhang, Yue Du

**Affiliations:** Department of Pediatrics, Shengjing Hospital of China Medical University, Shenyang, China

**Keywords:** overactive bladder, risk factors, atopic constitution, desloratadine, pediatrics

## Abstract

**Objective:** The present study aimed to analyze the risk factors correlated with overactive bladder (OAB), observe the effects of desloratadine in the treatment of OAB, and explore the correlation between OAB and atopic constitution in children.

**Methods:** Correlation and binary logistic regression analysis of the medical data from 447 children clinically diagnosed with OAB from June 2019 to June 2020 were conducted. The data included a history of urticaria, eczema, itchy skin, and allergic rhinitis or allergic cough. The OABSS scores before and after treatment with desloratadine were compared to evaluate the effectiveness of desloratadine for OAB.

**Results:** The risk factors for OAB in children included eczema, allergic rhinitis or allergic cough, itchy skin, and the levels of total blood IgE. Desloratadine was 96.5% effective in treating cases with risk factors including eczema, allergic rhinitis or allergic cough, and itchy skin. There existed statistical significance in the difference in OABSS scores before and after the treatment of desloratadine (*P* < 0.05).

**Conclusion:** OAB in children was correlated with atopic manifestations such as eczema, itchy skin, allergic rhinitis, or allergic cough. Desloratadine was safe and effective in the treatment of OAB in children with atopic manifestations.

## Introduction

Overactive bladder (OAB) is one of the most common lower urinary tract symptoms in children. OAB is defined by the International Children Continence Society (ICCS) as a type of urinary urgency, usually accompanied by symptoms of frequent urination and nocturia, with or without urge incontinence, and without urinary tract infection or other definite pathological changes (1). According to the symptom assessment tool for overactive bladder syndrome–overactive bladder symptom score (OABSS), urinary urgency ≥2 points and total score ≥3 points were diagnosed as OAB (2). Patients can be graded according to the total score: mild OAB is defined as a total score ≤5, moderate OAB is defined as a total score ≤11, and severe OAB is defined as a total score ≥12 (2). The etiology of OAB in children remains unclear. Clinical studies have shown that patients with OAB can sense bladder fullness, urge to urinate and pain more keenly than healthy people. OAB is characterized by anaphylaxis or enhanced bladder sensation, which is more pronounced in interstitial cystitis (3, 4). A study of 178 children with asthma by Soyer et al. found that asthmatic children younger than 6 years of age were at increased risk for frequent urination and urinary urgency, which occurred in 47.1% of the asthmatic children studied (5). A recent study of the relationship between asthma severity and lower urinary tract symptoms in adult men concluded that the asthmatic group had significantly higher urinary storage symptoms (frequent urination, urgency, and nocturia) and urination symptoms (tension, weakness, intermittent, and incomplete emptying) than the non-asthmatic group (6).

In recent years, it has been found clinically that some children with OAB have atopic constitutions concomitantly. The symptoms of OAB have improved with anti-allergic therapy. Therefore, the case data of children with OAB who visited the Department of Pediatric Nephrology and Rheumatology and the Pediatric Defecation and Urination Clinic in our hospital from June 2019 to June 2020 were analyzed and summarized.

## Materials and Methods

### Study Subjects

Children who were diagnosed as OAB at the Department of Pediatric Nephrology and Rheumatology and the Pediatric Defecation and Urination Clinic in Shengjing Hospital Affiliated to China Medical University from June 2019 to June 2020 were enrolled as the study subjects. All children were diagnosed in accordance with the OAB diagnostic criteria. Those with organic diseases such as urinary tract infections, stones, tumors, diabetes mellitus, and diabetes insipidus were excluded. There was no history of other complications or oral medications in any of the enrolled children. According to the OABSS scoring standard, the OAB score of mild, moderate, and severe OAB was conducted in all children. Urodynamic detections were performed in some cases. The control group consisted of children who had a physical examination in the Pediatric Development Clinic during the same period.

This study had been approved by the Ethics Committee of Shengjing Hospital Affiliated to China Medical University. This study was conducted in accordance with the declaration of Helsinki. Written informed consent was obtained from all participants.

### Methods

1. The results of children who underwent the urodynamic examinations were summarized, and any consistency between the clinical diagnosis and urodynamic results were compared.
2. The medical history data, including gender, age, history of eczema, urticaria, allergic rhinitis or allergic cough, itchy skin (eye rubbing, etc.), levels of total blood IgE and the results of urine routine test were collected. These were compared with the data from children who had a physical examination in the Pediatric Development Clinic during the same period to conduct risk factor analysis.
3. For all the children diagnosed with OAB, high-protein diets were restricted, the intake of irritative foods such as carbonated drinks was reduced, and prolonged urination time was encouraged. For those without improvement and with a history of eczema, urticaria, allergic rhinitis or allergic cough, itchy skin (eye rubbing, etc.), or elevated levels of total blood IgE, in addition to the continuation of high-protein diets restriction, oral administration of desloratadine was conducted. The dosages were as follows: In children aged 1–5, 1.25 mg of desloratadine was orally administered once daily. In children aged 6–11, 2.5 mg of desloratadine was given orally once daily. For those older than 12, 5 mg of desloratadine was orally administered once daily. The data of all children were recorded in the form of a urination diary, and the patients were followed up 2 weeks after treatment. The OABSS scoring was conducted before and after the desloratadinetreatment, and the scores were compared before and after the treatment.

### Statistical Analysis

The SPSS21.0 software was used for statistical analysis. *P* < 0.05 was considered statistically significant. The measurement data were expressed as means ± standard deviations, and the countable data were expressed by percentages (%). The *T*-test was adopted for the comparison of measurement data between groups, and the *x*^2^-test was used for the comparison of countable data between groups. The Chi-square test was used for univariate analysis of the factors that might affect OAB. Binary logistic regression analysis was adopted for the correlation analysis of the factors with statistically significant results in the univariate analysis.

## Results

### General Characteristics

A total of 447 cases with OAB were enrolled in the present study, including 232 males and 215 females with an average age of 6.40 ± 2.41. The age distribution of OAB patients is shown in Table 1. A total of 504 children who underwent physical examination were included in the control group, including 279 males and 225 females, with an average age of 6.59 ± 3.80. There was no significant difference between the two groups in gender composition and age (*P* > 0.05).

**Table 1:** Age of children with OAB.

**Ages**	**Number of patients in each OAB group**			**Total number of patients per age**
	**Mild OAB**	**Moderate OAB**	**Severe OAB**	
13 years old	0	0	2	2
12 years old	6	4	10	20
11 years old	7	2	10	19
10 years old	3	3	8	14
9 years old	12	3	15	30
8 years old	24	6	13	43
7 years old	31	5	14	50
6 years old	36	11	25	72
5 years old	55	14	11	80
4 years old	53	17	2	72

### Results of Urodynamics

In the present study, 447 cases with a clinical diagnosis of OAB were included. Cases with a history of renal disease, urinary tract infections, stones, tumors, and other diseases that might cause frequent urination and urgent urination were excluded. In the study, 148 patients underwent urodynamic examinations. The results showed 111 cases of detrusor overactivity, six cases of bladder hypersensitivity, five cases of detrusor external sphincter synergy, five cases of reduced urethral pressure, four cases of weakened detrusor function, one case of bladder outlet obstruction, and 16 cases without abnormality. Among these patients, 117 with detrusor hyperactivity + bladder hypersensitivity met the typical results in the urodynamics of OAB. Previous literature has reported (7, 8) that the reduction of urethral pressure and bladder outlet obstruction (6/148) are also important pathophysiological mechanisms of OAB. Thus, these urodynamic results were also consistent with the diagnosis of OAB. The above results indicated the urodynamic results were consistent with the clinical diagnosis (123/148). Therefore, the OABSS score could be applied to the diagnosis of OAB in children.

### Risk Factor Analysis

#### Univariate Analysis

The history of eczema, urticaria, allergic rhinitis or allergic cough, itchy skin (frequent eye rubbing, etc.), and levels of total blood IgE were included in the univariate analysis between the case group and the control group. The results showed that the risk factors for children with OAB included a history of eczema, urticaria, allergic rhinitis or allergic cough, itchy skin (frequent eye rubbing, etc.), and the levels of the total blood IgE (*P* < *0.01*). The detailed results are shown in Table 2.

**Table 2:** Univariate analysis of the risk factors in children with OAB.

**Grouping**	**OAB, *n* (%)**	**Control group, *n* (%)**	**t/Pearson's χ^**2**^**	***P***
Eczema			197.431	0.000
With	225/447(50.3)	46/504(9.1)		
Without	222/447(49.7)	458/504 (90.9)		
Urticaria			32.350	0.000
With	57/447 (12.8)	15/504 (3.0)		
Without	390/447 (87.2)	489/504 (97.0)		
Allergic rhinitis or allergic cough			89.848	0.000
With	137/447 (30.6)	35/504 (6.9)		
Without	310/447 (69.4)	469/504 (93.1)		
Itchy skin (Including frequent eye rubbing, etc)			74.503	0.000
With	143/447 (34.0)	48/504 (9.5)		
Without	304/447 (68.0)	456/504 (90.5)		
Level of total blood IgE	91.63 ± 164.963	22.76 ± 20.043	7.223	0.000

*The P-values in the results of the univariate analysis of eczema, urticaria, allergic rhinitis or asthma, itchy skin and the level of total blood IgE were all < 0.05, indicating the differences were statistically significant and further binary logistic regression analysis might be conducted*.

#### Binary Logistic Regression Analysis

**Figure 1:**
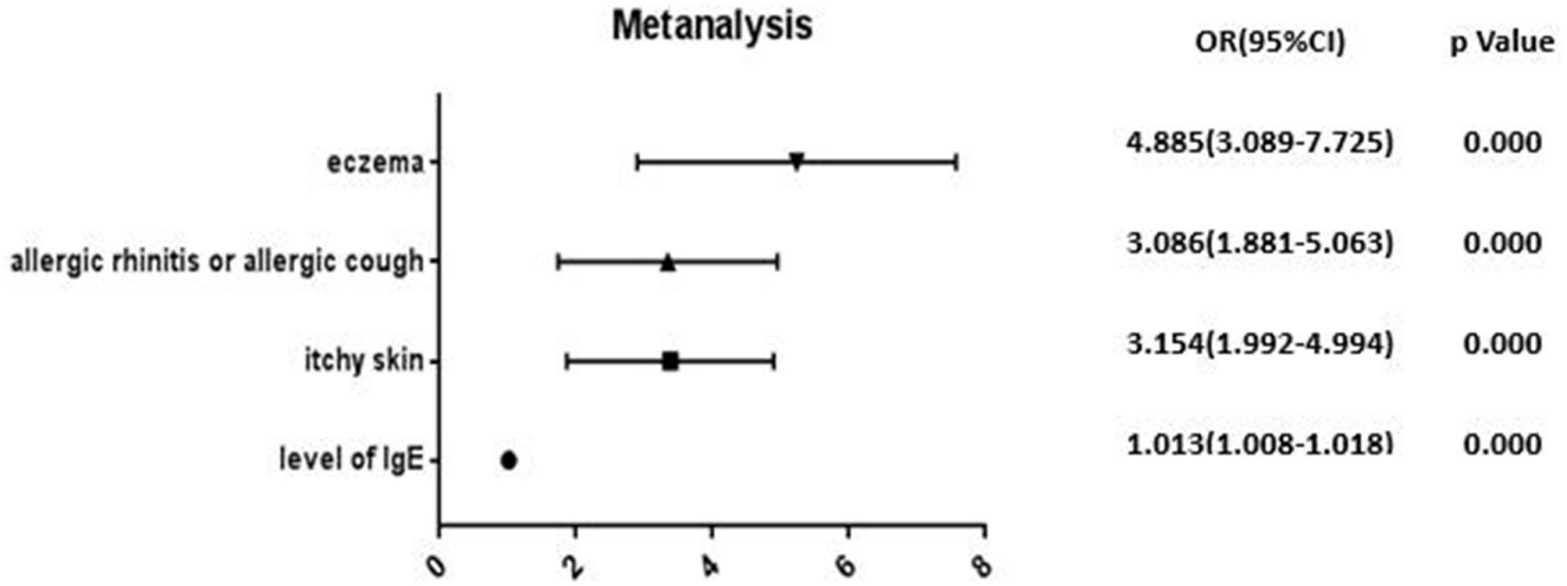
Binary logistic regression analysis of the risk factors in children with OAB.

The above-mentioned factors with significant results in the univariate analysis were introduced into the binary logistic regression model. The results of the binary logistic regression revealed that the risk factors for OAB in children included eczema (OR = 4.885), allergic rhinitis or allergic cough (OR = 3.086), itchy skin (including frequent eye rubbing) (OR = 3.154), and the levels of the total blood IgE (OR = 1.013). The details are illustrated in Figure 1.

#### OAB Grouping

The mild, moderate, and severe OAB were scored according to OABSS scoring standard using the OABSS scores, including daytime urination (0–2 points), nocturnal urination (0–3 points), urinary urgency (0–5 points), and urge incontinence (0–5 points. Those with a score ≤2 points were considered to be without symptoms of OAB, 3–5 points indicated mild OAB, 6–11 points indicated moderate OAB, and ≥12 points indicated severe OAB. The details are shown in Table 3.

**Table 3:** Grouping of children with OAB.

**Groups**	**Mild OAB**	**Moderate OAB**	**Severe OAB**	***P***
Total number of cases	257	76	114	
Gender (Male/Female)	135/122	34/42	63/51	0.347
Age	5.88 ± 2.09	5.98 ± 2.43	7.88 ± 2.46	0.000
OAB score	4.56 ± 0.72	8.28 ± 1.69	12.61 ± 0.63	0.000
Eczema (With/Without)	149/108	41/35	35/79	0.000
Urticaria (With/Without)	33/224	7/69	17/97	0.201
Allergic rhinitis or allergic cough (With/Without)	97/160	21/55	19/95	0.000
Itchy skin (Including frequent eye rubbing, etc) (With/Without)	111/146	31/45	1/113	0.000

*There were no statistical differences in gender among the mild, moderate and severe groups. The age in the severe group was older than those in the mild and moderate group. The P in the comparison of eczema, urticaria, allergic rhinitis or asthma and itchy skin was <0.05 among the mild, moderate and severe groups, indicating the existence of statistically significant differences. The proportion of children combined with atopic constitutions in the mild and moderate OAB group was higher than that in the severe OAB group*.

### Treatment

In 447 children with OAB, the symptoms of OAB were first controlled by controlling the intake of high-protein diets, reducing the intake of carbonated drinks and other stimulants, and encouraging prolonged urination. The symptoms of OAB were controlled in 131 cases. Among the remaining 316 cases, there were 169 cases of mild OAB, 57 of moderate OAB, and 90 of severe OAB. A total of 201 pediatric patients were treated with oral desloratadine due to a history of eczema, urticaria, allergic rhinitis or allergic cough, itchy skin, or elevated levels of total IgE. These included 154 cases of mild OAB and 47 cases of moderate OAB. The symptoms in 194 cases improved at follow-up 2–4 weeks after treatment (with the therapeutic efficacy of 96.5%). Comparison of the OABSS scores in children with OAB treated with desloratadine before and after treatment are shown in Table 4. No adverse effects occurred in these 201 cases. The proportion of children with atopic constitutions in the group of children with severe OAB was smaller than that in the group of children with mild or moderate OAB. Since only a small proportion of children had allergy-related symptoms in the severe OAB group, this group did not receive an oral administration of desloratadine. The details are illustrated in Table 3.

**Table 4:** Comparison of the OABSS scores before and after treatment of desloratadine in children with OAB.

	**Before treatment**	**After treatment**	***P***
Mild OAB	4.773 ± 0.554	0.760 ± 0.950	0.000
Moderate OAB	8.426 ± 1.885	1.660 ± 1.419	0.000
Total	5.627 ± 1.859	0.970 ± 1.140	0.000

*The P in the comparison of the OABSS scores before and after treatment of desloratadine in the mild and moderate group was <0.005, indicating the existence of statistically significant differences and effective therapeutic results*.

## Discussion

According to the definition by the ICCS, OAB is a concept based on the symptomatic diagnosis, which is a separate syndrome. Most patients with OAB exhibit urodynamics as overactive detrusor muscles. However, not all patients with OAB have urodynamics as overactive detrusors, and patients with urodynamics as overactive detrusors may not have symptoms of OAB. In the present study, 111 of 148 children with clinically diagnosed OAB showed urodynamic overactivity of the detrusors, accounting for 75% of the total patients. Additionally, 7.4% of the children showed urodynamic findings such as sensory bladder hypersensitivity, decreased urethral pressure, and obstruction of the bladder outlet. These findings were consistent with the results of the urodynamics in OAB. Therefore, we believe that the clinical diagnosis of OAB in children was highly consistent with the urodynamic findings. Children might be diagnosed and graded for OAB according to the criteria of the OABSS scoring.

OAB is common in children, and previous studies have shown that the prevalence of OAB in children ranges from 8 to 17.8% (9–11). OAB causes great inconvenience to life and learning in children, seriously affects the quality of life, and seriously influences mental health. Although previous studies have suggested a correlation between OAB in childhood and adulthood (12), the etiologies are not the same. The etiologies of OAB in children have been poorly studied. Some researchers have suggested that psychological factors (anxiety/depression), inappropriate diet, and deficiency of the micronutrient zinc are risk factors for OAB in children (13, 14). Our clinical observation found that the incidence of OAB was higher in children with atopic constitutions, but there is little research concerning this idea. Idiopathic daytime urinary frequency can be considered as mild OAB. Our previous study found that children with idiopathic daytime urinary frequency had high levels of total IgE and some atopic manifestations such as eczema, allergic rhinitis or cough, and itchy skin. Soyer et al. (5) also suggested that children with asthma had a higher incidence of frequent urination and urgent urination. The present study recorded a detailed history of eczema, urticaria, allergic rhinitis, allergic asthma, and itchy skin in children with OAB, and the detection of total IgE was conducted. The results indicated a correlation between OAB and atopic constitutions in children. Eczema, allergic rhinitis or allergic cough, itchy skin, and elevated levels of IgE might be risk factors for OAB in children. The mechanism might be that the mucous membrane of the lower urinary tract was highly reactive in children with atopic constitutions. When stimulated by known or unknown allergens, this might cause mucosal allergic reactions and lead to mucosal irritation or local muscle dynamics abnormalities, resulting in the occurrence of symptoms, including frequent urination and urgent urination.

The central nervous system that controls the bladder function, sensory nerves, bladder detrusor, and the urethral epithelium is involved in the development of OAB (15). Early in 1997, Hill et al. suggested that stimulation of histamine H1 receptors in the bladder smooth muscle triggers muscle contractions, which could be eliminated by the H1 blockers (16). In recent years, Stromberga et al. have found that histamine may cause an increase in the baseline tension in the uroepithelial and lamina propria (U&LP) and the detrusor layer and induce spontaneous contractions and an increase in the frequency of urethral epithelial and bladder contractions (17). The H1 receptors are expressed in tissues, including the smooth muscle, epithelial tissue, neurons, and various leukocytes (18). When histamine binds to the H1 receptor, it activates phospholipase C through G protein, producing inositol triphosphate (IP3) and diacylglycerol (DG). This increases the intracellular Ca2+ and activates protein kinase C, thereby contracting the smooth muscle (19). Meanwhile, mast cells can be visible in the uroepithelial, lamina propria, and smooth muscle layers of the bladder wall. The activation of mast cells causes the release of histamine, the binding of which to the receptors on the bladder wall causes inflammation and bladder sensitization (20). Moreover, histamine can also increase the afferent nerve's bladder sensitivity via H1 receptors, leading to overactive bladder contractions (21). Hypersensitivity of the bladder to the signals of the afferent nerve, bladder hypersensitivity, and contraction of the bladder smooth muscle can lead to the development of OAB. Desloratadine, as an H1 receptor antagonist, may selectively antagonize the H1 receptors while inhibiting the release of histamine from the mast cells, thereby relieving the symptoms of OAB. In the present study, 201 children were treated with oral desloratadine, and the improvement rate was 96.5% in 2–4 weeks, consistent with the above-mentioned studies. From a therapeutic perspective, these results confirmed that OAB in children might be correlated with atopic constitutions.

There were some limitations to the present study. Only children with mild or moderate OAB were treated with desloratadine and achieved good therapeutic effects in the study. However, the proportion of children with atopic constitutions in the group of children with severe OAB was smaller than that in the group of children with mild or moderate OAB. Since only a small proportion of children had allergy-related symptoms in the severe OAB group, no oral administration of desloratadine was given in this group. In the future, children in the severe OAB group may be treated with desloratadine, or an antihistamine and an M-blocker may be administered orally at the same time to treat both the cause and the effect. This may be more effective and allow a more comprehensive evaluation of the therapeutic effect.

In summary, the present study suggested that allergic rhinitis or allergic cough, eczema, itchy skin (eye rubbing, etc.), and elevated levels of total IgE were risk factors for the development of OAB in children. Mild to moderate OAB in children might be correlated with atopic constitutions. Oral administration of desloratadine could relieve the symptoms. It has a low incidence of adverse reactions in children with mild to moderate OAB and may provide new ideas and methods to benefit children with these related diseases in clinical practice.

## Data Availability Statement

The original contributions presented in the study are included in the article/supplementary material, further inquiries can be directed to the corresponding author/s.

## Ethics Statement

The studies involving human participants were reviewed and approved by ethics committee of Shengjing Hospital of China Medical University. Written informed consent to participate in this study was provided by the participants' legal guardian/next of kin. Written informed consent was not obtained from the individual(s), nor the minor(s)' legal guardian/next of kin, for the publication of any potentially identifiable images or data included in this article.

## Author Contributions

YZ and YD conceived the idea, conceptualized the study and drafted the manuscript. ZZ collected the data. LH and XW analyzed the data. KJ and SZ reviewed the manuscript. All authors read and approved the final draft. All authors contributed to the article and approved the submitted version.

## Conflict of Interest

The authors declare that the research was conducted in the absence of any commercial or financial relationships that could be construed as a potential conflict of interest.
